# Development and Evaluation of Three Indirect ELISA Methods Based on Novel Recombinant ASFV Antigens for Serological Detection of African Swine Fever

**DOI:** 10.3390/pathogens15090983

**Published:** 2026-09-16

**Authors:** Mo Zhou, Shengwei Ji, Li Zhang, Changchun Chen, Zhi Wu, Shinuo Cao, Shanyuan Zhu

**Affiliations:** 1 Jiangsu Key Laboratory for High-Tech Research and Development of Veterinary Biopharmaceuticals, Jiangsu Agri-Animal Husbandry Vocational College, Taizhou 225306, China; 2 Department of Veterinary Medicine, Agriculture College of Yanbian University, Yanji 133000, China

**Keywords:** African swine fever virus (ASFV), serological detection, indirect ELISA, recombinant antigen

## Abstract

African swine fever (ASF) poses a catastrophic and ongoing threat to the global swine industry. The rapid intercontinental spread of ASF, compounded by the absence of licensed vaccines and the structural complexity of African swine fever virus (ASFV), renders disease control and eradication extraordinarily challenging. Serological diagnosis is central to long-term ASF surveillance and control, with current assays predominantly based on well-validated antigens such as p30 (CP204L), p54 (E183L), and p72 (B646L), which form the backbone of OIE/WOAH-recommended testing. To expand and complement this established diagnostic repertoire with additional, less-explored targets. In this study, three ASFV proteins, B169L, A151R, and KP177R, were identified through systematic bioinformatic screening and successfully expressed using a prokaryotic expression system. Three individual indirect ELISA methods were subsequently established, optimized, and comprehensively evaluated. All three assays demonstrated high specificity, with no cross-reactivity detected against antisera to PRRSV, CSFV, or PCV2. Intra-assay coefficients of variation (CVs) were below 6% and inter-assay CVs were below 8%, confirming excellent repeatability and reproducibility. These assays provide sensitive, specific, and practically deployable tools that expand the available options for ASF serological surveillance and may serve as useful complements to existing diagnostic methods in epidemiological investigations of this devastating disease.

## 1. Introduction

African swine fever (ASF) is a devastating and highly contagious viral disease affecting both domestic and wild pigs. Clinically, it manifests as high fever, anorexia, and extensive hemorrhaging in the skin and internal organs, culminating in a variable case fatality rate that depends on the virulence of the infecting strain, with severe infections typically causing death within 2–10 days post-infection. The causative agent, African swine fever virus (ASFV), is a large, enveloped, double-stranded DNA virus and the sole member of the family Asfarviridae. Its complex molecular architecture, remarkable environmental stability, and capacity for transmission via direct contact, contaminated feed, and soft tick vectors collectively render ASFV one of the most significant threats to global animal health [1,2,3,4].

ASF has inflicted unprecedented economic losses on the global swine industry, severely disrupted international trade, and raised widespread concerns over food security [5]. Serological diagnosis has emerged as an indispensable component of long-term ASF surveillance and control programs [5,6]. Unlike nucleic acid–based detection methods, serological assays can identify animals that have recovered from infection or undergone subclinical exposure, given that ASFV-specific antibodies persist for extended periods. Among available serological platforms, enzyme-linked immunosorbent assays (ELISAs) are particularly well suited for large-scale screening owing to their high throughput, operational simplicity, and cost-effectiveness [6,7,8,9].

The current landscape of ASFV serological diagnostics is anchored by well-validated assays based on classical antigens. Most existing ELISA-based assays rely on well-established structural proteins, primarily p72 (B646L), p54 (E183L), and p30 (CP204L) [10,11,12,13]. These antigens are well validated, widely used, and form the backbone of OIE/WOAH-recommended serological diagnosis. In parallel, expanding the repertoire of available antigens may offer complementary value and further strengthen diagnostic coverage in specific epidemiological contexts. For example, p30 is an early-phase protein expressed predominantly during the initial stages of infection, and anti-p30 antibody titers may wane in a subset of convalescent and chronically infected animals [14]. Additionally, while p72 is highly conserved across genotypes and serves as the basis for ASFV genotype classification [15,16], independent comparative evaluations of commercial ELISAs have noted occasional inter-kit discordance and variable sensitivity against certain field strains or low-titer samples [17]. Moreover, antibodies against p30, p54, and p72 are not uniformly elicited in all infected animals [18]. These observations motivate the exploration of complementary antigens that could diversify detection capabilities alongside, rather than replace, existing proven assays.

In this context, we selected three alternative proteins—B169L, A151R, and KP177R- as complementary candidates based on their distinct biological properties, immunogenic potential, and broad conservation across ASFV genotypes. B169L is located within the central conserved region of the ASFV genome (~80–99 kb) and is transcribed in the leftward direction. It encodes a 159–174 amino acid double-transmembrane envelope protein implicated in virion morphogenesis and membrane association. Recent studies have further identified B169L as a functional viroporin that forms Ca^2+^-permeable channels and localizes to the inner envelope of virions and the endoplasmic reticulum, playing an essential role in proinflammatory responses and viral replication. Comparative sequence analysis of all available B169L entries in GenBank, encompassing 12 of the 24 p72 genotypes (I, II, III, IV, V, VII, VIII, IX, X, XI, XV, and XX), revealed pairwise amino acid identities ranging from 90.9% to 99.4%. The N-terminus and both transmembrane domains are nearly completely conserved, whereas variation is restricted to the C-terminal proline-rich repeat tail. Notably, B169L is pseudogenized in the genotype XX/XXII lineage, and no sequences are currently available for the remaining 11 genotypes, as complete genomes have yet to be determined. B169L has repeatedly been identified among the most immunoreactive ASFV proteins in proteome-wide immunoscreening studies, indicating strong B-cell antigenicity during natural infection. This robust seroreactivity, combined with its high inter-genotype conservation, underscores its potential as a candidate antigen for ASFV serological detection [19].

A151R is located in the left-central region of the ASFV genome (~31.5–52 kb, depending on the strain) and is transcribed in the rightward direction. It encodes a 150–153 amino acid soluble nonstructural protein that is expressed during both early and late stages of the viral replication cycle, with predominant expression in the late phase. Functionally, A151R has been implicated in viral DNA metabolism and repair, as well as in the modulation of host innate immunity, including the suppression of type I interferon signaling. Comparative sequence analysis of all available A151R entries in GenBank, encompassing 13 of the 24 p72 genotypes, revealed pairwise amino acid identities ranging from 64.0% to 100%, indicating that A151R is substantially more variable than structural proteins such as B169L. Notably, no A151R sequences are currently available for the remaining 11 genotypes, as complete genomes have yet to be determined for these lineages. At the molecular level, A151R contains a conserved thioredoxin-like CXXC motif and interacts with the nonstructural protein B119L as part of the redox pathway required for disulfide bond formation in the structural protein E248R. This interaction is essential for viral replication and contributes to virulence modulation [20]. Because A151R is absent from the mature virion, anti-A151R antibodies are elicited only by active viral replication, making this protein an attractive early serological marker for monitoring active infection.

KP177R is located within the left variable region near the left terminus of the ASFV genome (~2.3–5.2 kb, depending on the strain) and is transcribed in the rightward direction. It encodes a 169–177 amino acid cysteine-rich virion structural protein, also designated p22, that is expressed in the late phase of infection and bears a predicted N-terminal signal peptide. The protein is localized to the inner membrane of the virion and is transiently displayed on the surface of infected cells. Although its precise function remains incompletely characterized, KP177R has been implicated in virion assembly and morphogenesis, and its cysteine residues are absolutely conserved across all available sequences. Notably, KP177R has recently been shown to interact with host partners involved in several cellular pathways, whereas deletion of the gene does not significantly alter viral replication in vitro or virulence in vivo. Comparative sequence analysis of all available KP177R entries in GenBank, encompassing 13 of the 24 p72 genotypes, revealed pairwise amino acid identities ranging from 80.5% to 100%, with 70.0% of alignment columns fully conserved. These sequences resolve into two macro-clades. No KP177R sequences are currently available for the remaining 11 genotypes, as complete genomes have yet to be determined. Despite its non-essential role in viral replication and virulence, KP177R is highly conserved among ASFV isolates and has demonstrated strong seroreactivity in diagnostic studies, underscoring its potential as a candidate antigen for ASFV serological detection [21,22].

In the present study, we systematically expressed and evaluated these three alternative proteins for their individual diagnostic potential. On this basis, we subsequently developed separate indirect ELISA methods targeting each of these three proteins, as an expansion of the available toolkit for the serological detection and epidemiological surveillance of ASF.

## 2. Materials and Methods

### 2.1. Ethics Statement

No experimental infection of animals was conducted in the present study. The ASFV-positive sera used for assay validation were purchased as certified reference materials from the National Institutes for Food and Drug Control (NIFDC, Beijing, China). The negative sera were collected from clinically healthy pigs maintained on ASFV-free farms. The collection and use of these negative serum samples were conducted in strict accordance with the Regulations for the Administration of Affairs Concerning Experimental Animals issued by the Ministry of Science and Technology of China, and the protocol was reviewed and approved by the Scientific Ethics Committee of Jiangsu Agri-animal Husbandry Vocational College (Approval No. JSAHVC-2024-69).

### 2.2. In Silico and Bioinformatics Analysis of Candidate Antigens

Candidate ASFV diagnostic antigens were identified through systematic bioinformatic interrogation of the ASFV genome (Georgia 2007/1). The coding sequences analyzed in this study were as follows: B169L, CAD2068439.1; A151R, CAD2068398.1; and KP177R, CAD2068339.1.

**Figure 1:**
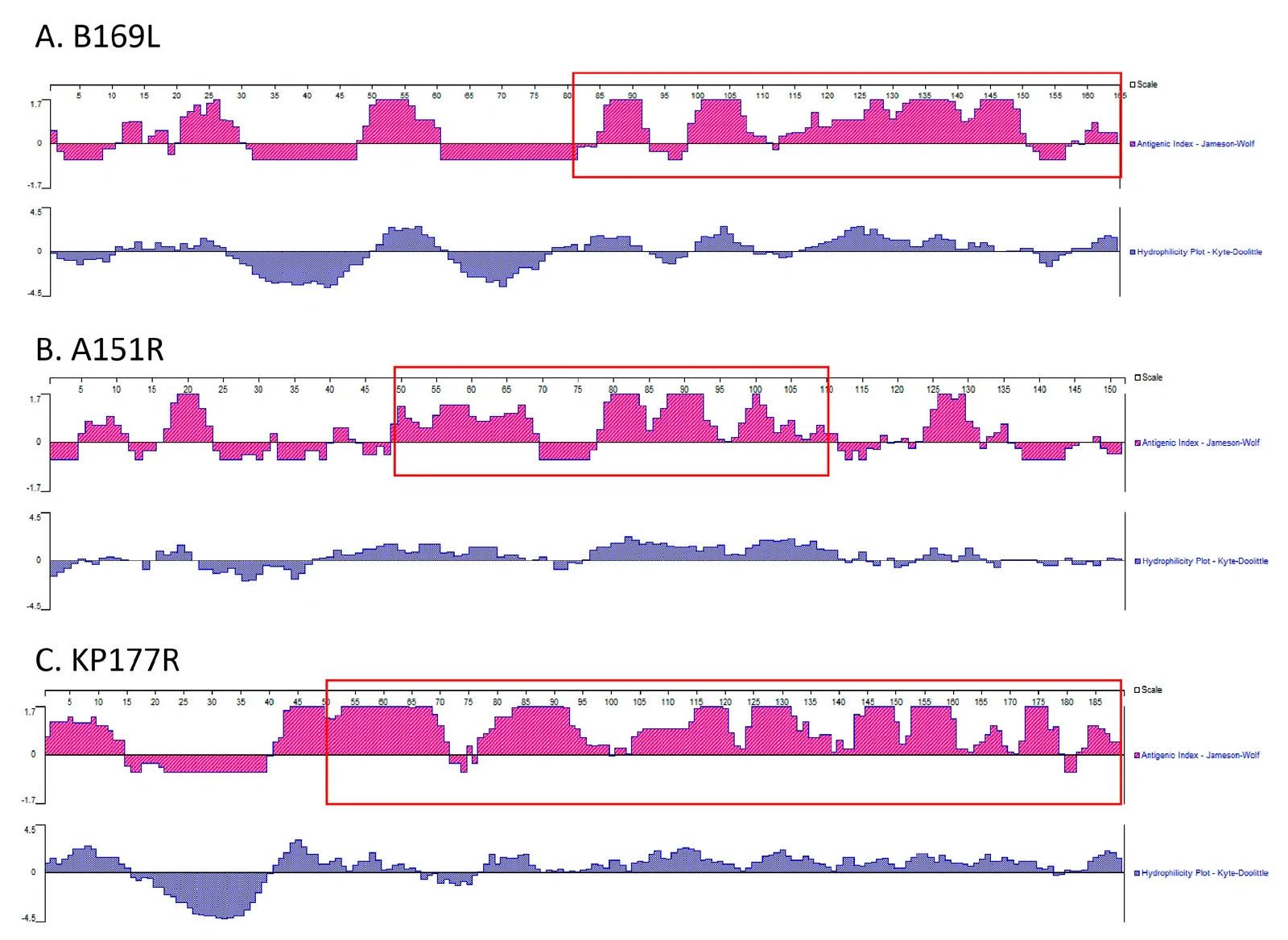
Bioinformatic profiling of ASFV−B169L (**A**), ASFV−A151R (**B**), and ASFV−KP177R (**C**). The upper panels show the Jameson−Wolf antigenic index (pink), and the lower panels show the Kyte-Doolittle hydropathy plots (blue). Red boxes indicate the protein regions selected for recombinant expression: amino acids 81−164 of B169L (**A**), 50−110 of A151R (**B**), and 38−177 of KP177R (**C**).

The physicochemical properties, signal peptides, and transmembrane topologies of the three candidate proteins were evaluated using ProtParam (https://web.expasy.org/protparam/, accessed on 9 January 2026), SignalP 5.0 (https://services.healthtech.dtu.dk/services/SignalP-5.0/ (accessed on 9 January 2026), TMHMM 2.0 (https://services.healthtech.dtu.dk/services/TMHMM-2.0/ (accessed on 9 January 2026), and AlphaFold2 (https://alphafold.ebi.ac.uk/ (accessed on 9 January 2026). Truncation boundaries for recombinant expression were defined based on the resulting hydrophobicity profiles, epitope density distributions, and predicted solubility, with the aim of excluding hydrophobic or transmembrane regions while retaining immunodominant, hydrophilic fragments (Figure 1).

### 2.3. Gene Cloning and Protein Expression

The coding sequences of all three target genes were codon-optimized for expression in *E. coli*, chemically synthesized, and individually cloned into the pET-28a(+) expression vector (Novagen (Merck, Darmstadt, Germany)), which appends an N-terminal hexahistidine (His_6_) tag to the recombinant proteins. The resulting recombinant plasmids were transformed into *E. coli* BL21(DE3) competent cells from TransGen Biotech (Beijing, China), and protein expression was induced with 0.5 mM isopropyl β-D-1-thiogalactopyranoside (IPTG) (Beyotime Biotechnology, Shanghai, China) at 30 °C for 6 h. Cells were harvested by centrifugation, lysed by sonication, and the His-tagged fusion proteins were purified by Ni-NTA affinity chromatography (Beyotime Biotechnology, Shanghai, China). Protein purity and identity were subsequently confirmed by SDS-PAGE analysis.

### 2.4. Serum Samples

Swine serum samples were assembled to evaluate the indirect ELISAs. The ASFV-positive sera were obtained from the National Institutes for Food and Drug Control (NIFDC, Beijing, China) as certified ASFV antibody reference materials. These are characterized, quality-controlled materials derived from domestic pigs experimentally infected with ASFV genotype II; they are not wild boar samples, nor are they field outbreak sera. ASFV-negative sera were obtained from clinically healthy pigs maintained on ASFV-free farms and confirmed negative by qPCR and a commercial ELISA kit. To assess the specificity, additional sera positive for heterologous porcine pathogens were included. The exact panel composition evaluated for each antigen was as follows: B169L, 12 ASFV-positive, 30 ASFV-negative, 2 CSFV-positive, 5 PRRSV-positive, and 2 PCV2-positive; A151R, 12 ASFV-positive, 30 ASFV-negative, 3 CSFV-positive, 3 PRRSV-positive, and 3 PCV2-positive; and KP177R, 12 ASFV-positive, 30 ASFV-negative, 3 CSFV-positive, 3 PRRSV-positive, and 3 PCV2-positive. Owing to the limited availability of validated ASFV-positive samples at the time of this preliminary study, the assays were restricted to genotype II sera. This constraint precludes evaluation of cross-genotype reactivity, and the claim of broader detection capability for these antigens therefore remains unproven within the scope of the present dataset.

### 2.5. Western Blotting Analysis

To confirm the antigenicity of the purified recombinant proteins, proteins were resolved by SDS-PAGE and electrophoretically transferred onto PVDF membranes (Beyotime Biotechnology, Shanghai, China). Membranes were blocked with 5% skimmed milk (Beyotime Biotechnology, Shanghai, China) powder in PBST for 2 h at room temperature and subsequently incubated overnight at 4 °C with ASFV-positive or ASFV-negative swine sera diluted 1:200 in 5% skimmed milk-PBST. After washing, membranes were incubated with horseradish peroxidase (HRP)-conjugated goat anti-swine IgG secondary antibody (Beyotime Biotechnology, Shanghai, China) diluted 1: 5000, and immunoreactive bands were visualized using DAB substrate solution (Beyotime Biotechnology, Shanghai, China).

### 2.6. Screening of Optimal Coating Antigen Concentrations

To determine optimal assay conditions for each antigen, ELISA plates were coated with 100 µL of recombinant protein at varying concentrations (1.5–6.0 µg/mL) in carbonate buffer (0.05 mol/L, pH 9.6) and incubated overnight at 4 °C. After washing, plates were blocked with 5% skimmed milk powder for 12 h at 4 °C. Serum samples diluted 1:100 in PBST were added and incubated at 37 °C for 1 h, after which the secondary antibody (HRP-conjugated goat anti-swine IgG, diluted 1:5000) was applied and plates incubated at 37 °C for 1 h. Color development was performed with TMB substrate (Beyotime Biotechnology, Shanghai, China) for 15 min, and the reaction was terminated by addition of 2 M H_2_SO_4_. Absorbance was measured at 450 nm (OD_450_) with a microplate reader (Thermo Fisher Scientific, Waltham, MA, USA). The optimal coating concentration was defined as the condition yielding the highest positive-to-negative (P/N) OD450 ratio.

### 2.7. Repeatability Assay

Intra-assay repeatability was evaluated by testing five replicates per serum sample within a single microplate. Inter-assay repeatability was assessed by analyzing the identical panel across three independent plates performed on separate days. Coefficients of variation (CVs) were calculated for each sample under both intra- and inter-assay conditions. Predefined acceptability criteria were CV < 10% for intra-assay and CV < 15% for inter-assay variation.

### 2.8. Determination of Diagnostic Performance and Cut-Off Value

The cut-off value for each ELISA was defined as the mean OD450 of ASFV-negative control sera plus three standard deviations (Mean + 3SD). All ASFV-positive and -negative samples were tested in duplicate. Samples with OD450 values at or above the cut-off were classified as seropositive; those below were classified as seronegative. Diagnostic sensitivity and specificity were calculated from the classification results.

### 2.9. Specificity Assay

The specificity of each optimized ELISA was assessed using the fully characterized serum panel described in Section 2.4. All ASFV-negative and heterologous pathogen-positive sera were tested in duplicate under the respective optimized assay conditions. OD450 values were compared against the cut-off threshold established for each antigen. Samples with OD450 values below the cut-off were classified as negative. Specificity was calculated as the proportion of true-negative samples (ASFV-negative and heterologous pathogen-positive sera correctly classified as negative) among all non-ASFV sera tested. In parallel, ASFV-positive sera included in the panel were run concurrently to enable evaluation of diagnostic sensitivity and cut-off performance. We acknowledge that the limited panel size, particularly for some heterologous pathogen groups, constrains statistical power; accordingly, all specificity claims are explicitly qualified as applying within the tested sample panel only.

### 2.10. Statistical Analysis

Data were analyzed using GraphPad Prism 9.0 and SPSS 26.0, with results expressed as mean ± SD and significance set at *p* < 0.05. Cut-off values were determined as the mean OD450 of ASFV-negative control sera plus three standard deviations (Mean + 3SD). Diagnostic sensitivity and specificity are reported with 95% confidence intervals calculated by the Clopper-Pearson exact method. Differences among groups were evaluated by one-way ANOVA followed by Tukey’s post hoc test.

## 3. Results

### 3.1. Bioinformatic Characterization of Candidate Antigens

The coding sequences of B169L, A151R, and KP177R were translated into protein sequences comprising 164, 151, and 177 amino acids, respectively. Physicochemical properties were predicted using ProtParam: B169L, A151R, and KP177R exhibited theoretical molecular weights of 18.32 kDa, 17.78 kDa, and 20.15 kDa, and isoelectric points (pI) of 6.27, 7.69, and 8.00, respectively. Subcellular localization and membrane topology were further analyzed using SignalP 5.0 and TMHMM 2.0. SignalP 5.0 predicted no signal peptide cleavage sites in any of the three proteins (Figure 1). TMHMM 2.0 analysis revealed that A151R lacks transmembrane helices (expected number of amino acids in transmembrane helices, Exp AA: 0.069). In contrast, B169L was predicted to contain two transmembrane helices spanning residues 26-48 and 61-79 (Exp AA in TMHs: 40.33; N-terminal inside probability, N-in: 0.116), whereas KP177R harbors a single transmembrane helix at residues 7-29 (Exp AA in TMHs: 21.93; N-in: 0.450).

**Figure 2:**
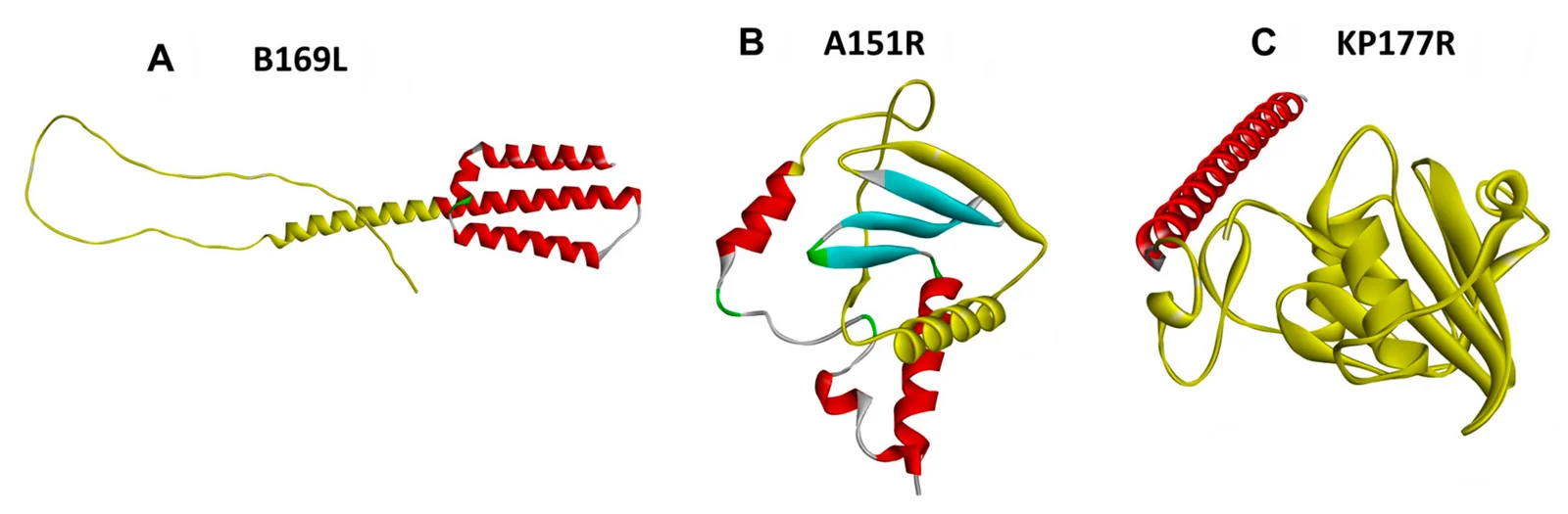
Three-dimensional structures of ASFV-B169L (**A**), ASFV-A151R (**B**), and ASFV-KP177R (**C**) predicted by AlphaFold2. The recombinant antigen expression regions are highlighted in yellow.

Based on these predictions, truncation boundaries were defined for B169L and A151R. The expressed regions and corresponding codon-optimized nucleotide and amino acid sequences are summarized in Table 1. The three-dimensional structures of B169L, A151R, and KP177R were subsequently modeled using AlphaFold2. The predicted spatial architectures, together with the recombinant antigen expression regions (highlighted in yellow), are presented in Figure 2A, Figure 2B, and Figure 2C, respectively.

### 3.2. Prokaryotic Expression, Purification, and Immunogenicity of Recombinant Proteins

**Figure 3:**
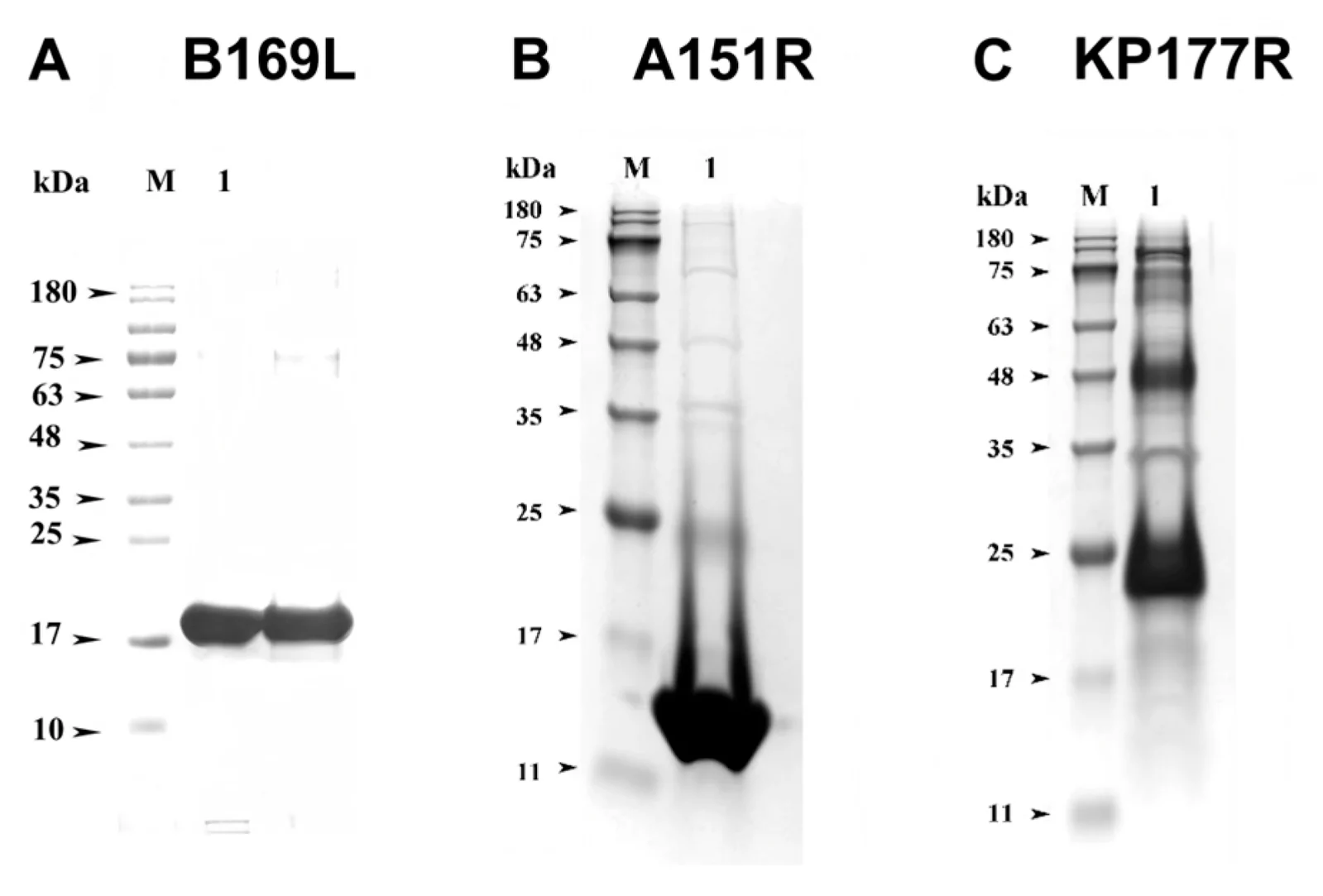
SDS-PAGE analysis of purified recombinant ASFV-B169L (**A**), ASFV-A151R (**B**), and ASFV-KP177R (**C**). Lane M, protein molecular weight marker (kDa); Lane 1, purified recombinant protein.

**Figure 4:**
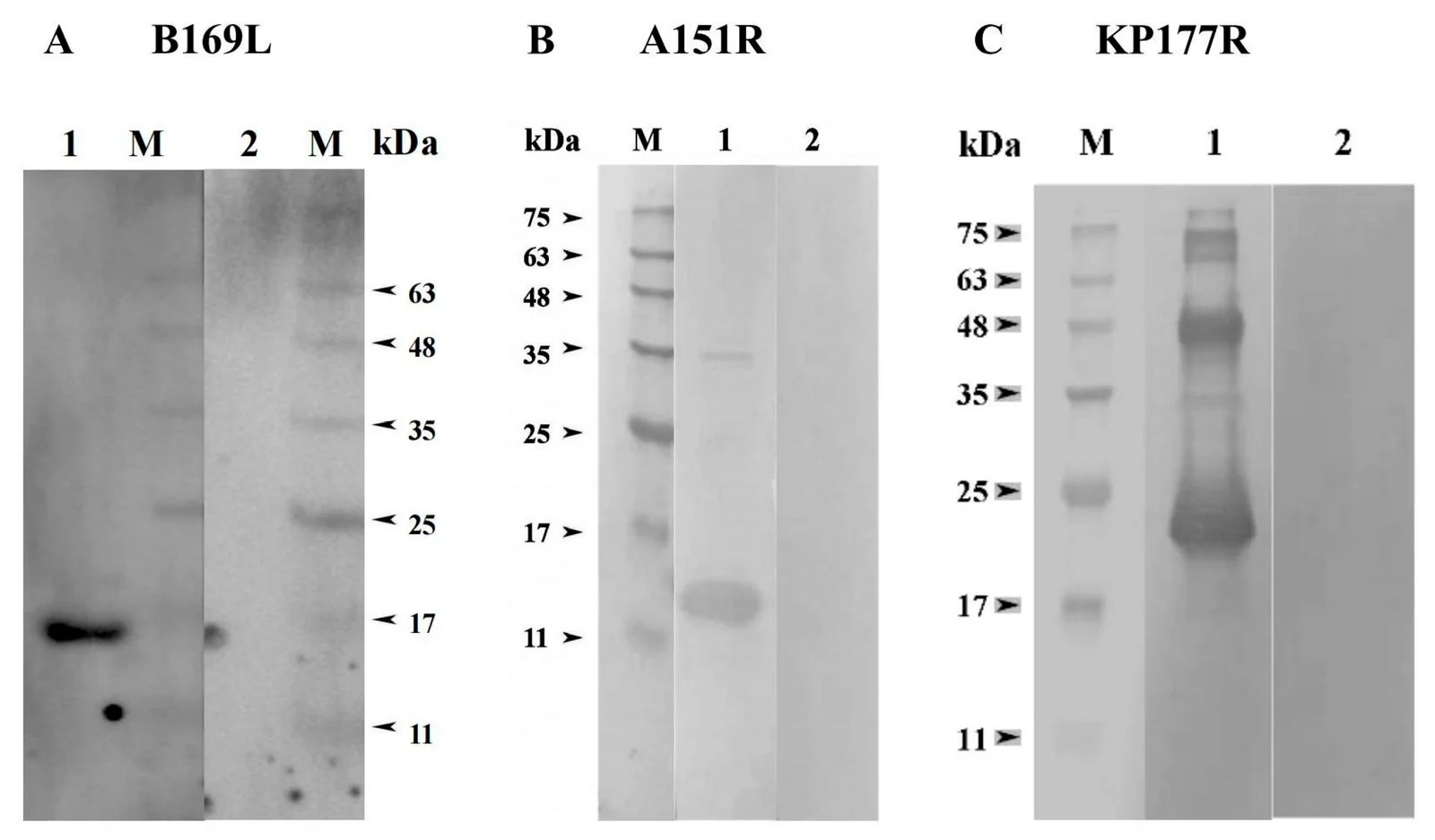
Western blot analysis of the immunoreactivity of recombinant ASFV-B169L (**A**), ASFV-A151R (**B**), and ASFV-KP177R (**C**). Purified His-tagged proteins were separated by SDS-PAGE, transferred to PVDF membranes, and probed with ASFV-positive swine serum (Lane 1) or ASFV-negative swine serum (Lane 2), followed by HRP-conjugated goat anti-swine IgG. Lane M, protein molecular weight marker (kDa).

Recombinant proteins B169L, A151R, and KP177R were efficiently expressed in *E. coli* BL21(DE3) following IPTG induction and purified to high homogeneity by Ni-NTA affinity chromatography. SDS-PAGE analysis revealed distinct bands at the expected molecular weights: approximately 16.87 kDa for ASFV-B169L, 10.68 kDa for ASFV-A151R, and 23.41 kDa for ASFV-KP177R (Figure 3A–C). Western blot analysis further confirmed the immunoreactivity of the purified proteins: all three recombinant antigens were specifically recognized by ASFV-positive swine serum, whereas no specific reactivity was detected with ASFV-negative swine serum (Figure 4A–C), demonstrating that the purified recombinant proteins retain the antigenic epitopes of native ASFV and are suitable as diagnostic antigens. Notably, a faint higher-molecular-weight band (~47 kDa, approximately twice the monomer size) was also observed for ASFV-KP177R protein. KP177R (p22) contains multiple absolutely conserved cysteine residues, and this faint band corresponds to SDS-resistant dimers formed via intermolecular disulfide bonds that are not fully reduced during sample preparation and/or incomplete denaturation, a well-documented behavior of cysteine-rich recombinant proteins. Densitometric quantification confirmed that the monomer accounts for ≥90% of total purified protein. Regarding diagnostic accuracy, the coating antigen comprises the total purified preparation; the dimeric form retains the same epitopes and is equally immunoreactive in Western blot. Consequently, this minor dimer population does not affect diagnostic accuracy, as evidenced by the concordant classification, absence of cross-reactivity, and inter-assay CVs below 8%.

### 3.3. Optimization of ELISA Conditions

ELISA conditions were systematically optimized for all three antigens. Optimal analytical performance was achieved when plates were coated with recombinant protein at a final concentration of 2.0 µg/mL (100 µL per well, equivalent to 200 ng per well) in carbonate buffer (0.05 mol/L, pH 9.6) and incubated overnight at 4 °C. Serum samples diluted 1:100 in PBST were then added and incubated at 37 °C for 1 h, followed by incubation with HRP-conjugated goat anti-swine IgG secondary antibody (diluted 1:5000) at 37 °C for 1 h.

### 3.4. Specificity of the ELISA Assays

**Figure 5:**
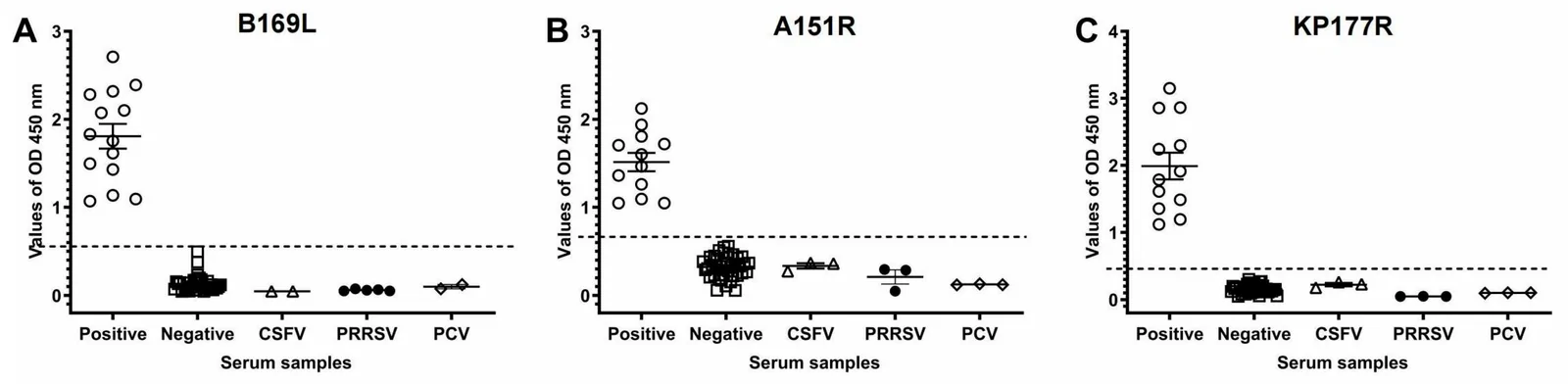
Determination of cut-off values and specificity for the indirect ELISAs based on recombinant ASFV-B169L (**A**), ASFV-A151R (**B**), and ASFV-KP177R (**C**). Individual OD450 values are plotted for each serum category under the respective optimized conditions. (**A**) B169L assay: 12 ASFV-positive, 30 ASFV-negative, 2 CSFV-positive, 5 PRRSV-positive, and 2 PCV2-positive sera; the dashed horizontal line indicates the cut-off value of 0.58, calculated as the mean OD450 of ASFV-negative sera plus 3SD. (**B**) A151R assay: 12 ASFV-positive, 30 ASFV-negative, 3 CSFV-positive, 3 PRRSV-positive, and 3 PCV2-positive sera; the cut-off value was 0.713. (**C**) KP177R assay: 12 ASFV-positive, 30 ASFV-negative, 3 CSFV-positive, 3 PRRSV-positive, and 3 PCV2-positive sera; the cut-off value was 0.353. All ASFV-positive samples registered OD450 values above the respective cut-offs, whereas all ASFV-negative and heterologous-pathogen-positive samples fell below them, confirming the high specificity of the three assays within the tested sample panel.

The specificity of each assay was evaluated using antisera against CSFV, PRRSV, and PCV2. All non-ASFV sera yielded OD450 values below the established cut-off thresholds (Figure 5), demonstrating that none of the three ELISAs exhibited detectable cross-reactivity with antibodies to these heterologous porcine pathogens. These results confirm the specificity of the developed assays for ASFV antibody detection.

### 3.5. Repeatability of the ELISA Assays

Intra- and inter-assay repeatability were assessed using positive and negative serum panels. As presented in Table 2, for all three established ELISAs, intra-assay CVs varied between 1.05% and 5.63%, with the highest value less than 6% overall. Inter-assay CVs ranged from 2.30% to 7.68%, and the maximum value was obtained from sample P1 tested via the B169L ELISA, with all values below 8%. Collectively, both intra- and inter-assay CVs complied with our predetermined acceptance thresholds (intra-assay CV < 10%; inter-assay CV < 15%).

### 3.6. Determination of Cut-Off Values and Diagnostic Performance

Cut-off values were calculated as the mean OD450 of ASFV-negative control sera plus three standard deviations (Mean + 3SD), yielding cut-offs of 0.58 (B169L), 0.713 (A151R), and 0.353 (KP177R). Under these thresholds, all ASFV-positive sera exceeded the cut-off values while all ASFV-negative and heterologous pathogen-specific sera fell below it (Figure 5). These statistically validated thresholds confirm that the three ELISAs are robust and discriminatory serological tools for detecting ASFV-specific antibodies.

## 4. Discussion

African swine fever (ASF) remains one of the most devastating threats to the global swine industry, and its rapid transcontinental transmission underscores an urgent demand for robust, accurate, and scalable diagnostic tools [13,23,24]. Current ASFV serological diagnostics are anchored by well-validated assays based on the classical structural proteins p72, p54, and p30, which constitute the backbone of OIE/WOAH-recommended serological diagnosis and have proven effective in large-scale field application [14]. In an effort to expand and complement this established diagnostic repertoire with additional, less-explored antigen targets, the present study identified and validated three additional recombinant antigens, ASFV-A151R, B169L, and KP177R, as complementary serological targets. These candidates were selected based on high genotypic conservation, strong predicted antigenicity, and favorable expression characteristics [25,26,27,28], and can be efficiently produced in *E. coli* at reduced cost. These findings align with and extend prior reports on the diagnostic utility of ASFV alternative antigens. KP177R (p22), in particular, has been repeatedly identified as a dominant seroreactive protein in proteome-wide immunoscreening studies and has previously been evaluated as a recombinant antigen in indirect ELISA and Luminex-based multiplex platforms [21,22,29]. The robust seroreactivity observed here for p22 is consistent with these earlier reports, reinforcing its established value as a complementary diagnostic target. B169L and A151R, while less extensively characterized in the serological literature, have similarly emerged among the most immunoreactive ASFV proteins in recent proteomic and protein microarray analyses [19,20], supporting their potential contribution alongside classical structural antigens.

The biological basis for selecting these particular proteins lies in their distinctive immunogenic properties. Although ASFV encodes more than 150 proteins, only a small fraction elicits detectable humoral responses in infected pigs. The proteins pB169L, pA151R, and p22 (encoded by KP177R) represent three functionally distinct examples that consistently provoke antibody production. pB169L is an inner-envelope transmembrane protein essential for virion morphogenesis and functions as a viroporin that perturbs intracellular calcium homeostasis, thereby activating the NLRP3 inflammasome and promoting IL-1β secretion [30]. pA151R is a non-structural thioredoxin-like oxidoreductase that supports viral protein folding during replication while antagonizing innate immunity by degrading the E3 ligase TRAF6 and suppressing TBK1 phosphorylation and type I interferon production [19]. p22 is an early-expressed, inner-envelope-associated protein involved in virus factory organization and genome replication, and is consistently ranked among the dominant seroreactive antigens of ASFV [30,31].

Several converging features explain the humoral immunogenicity of these three proteins. The pB169L and p22 are incorporated into virions or viral factory membranes, and the massive viremia characteristic of ASFV amplifies their extracellular abundance, whereas pA151R is abundantly expressed early and released upon lysis of infected monocytes/macrophages, the virus’s primary target cells and professional antigen-presenting cells. Moreover, membrane-exposed domains of pB169L and p22 are directly recognizable by B-cell receptors, and ASFV-driven apoptotic body dissemination efficiently delivers concentrated membrane antigens to secondary lymphoid organs. Furthermore, they offer a temporal advantage: p22 and pA151R are expressed early, before viral immune-evasion machinery fully paralyzes antigen presentation. B169L-driven inflammasome activation and A151R-associated ferroptotic cell death provide danger signals that facilitate B-cell activation and T-cell help.

In contrast, most ASFV proteins remain serologically silent. Proteomic atlases show that numerous virion components are encapsulated within the core shell and nucleoid, physically shielded from B-cell recognition [32]. Many non-structural proteins are expressed at low levels or late in infection, after viral immunosuppressors (e.g., MGF360/505 family members, A238L, DP96R) have dismantled interferon signaling, antigen presentation, and co-stimulation. Furthermore, ASFV-induced bystander lymphocyte apoptosis depletes the B-cell and Tfh compartments, while many proteins lack porcine SLA-II-binding epitopes required for class switching. Consequently, humoral immunogenicity in ASFV emerges only when high antigen dose, extracellular accessibility, early expression, inflammatory context, and T-cell help coincide, criteria that B169L, A151R, and p22 fulfill based on their biological properties and documented seroreactivity [31].

The three antigen-based ELISAs demonstrated high specificity, with no cross-reactivity against PRRSV, CSFV, or PCV2 antisera, supporting reliable differential diagnosis. Leveraging the durability of ASFV-specific humoral immunity, these assays enable identification of seropositive recovered pigs and are readily scalable for large-scale screening in both endemic and ASF-free regions. Because individual animals may mount variable antibody responses to different viral proteins, incorporating additional antigens with distinct epitope repertoires, such as the underexplored targets characterized here, into a multi-antigen testing strategy could potentially broaden the overall scope of serological detection. Such diversification of the antigen panel may help capture infections that might otherwise be missed when relying on a single antigen, particularly in the context of heterogeneous host immune responses or variable antibody kinetics [7,8,33,34]. Nevertheless, it is important to emphasize that commercial multi-antigen ELISAs are already available, and the data presented herein do not demonstrate that these new antigens overcome any specific limitation of, or provide superior performance to, existing diagnostic tools.

It is noteworthy that the A151R ELISA exhibited a relatively high cut-off value (0.713). This reflects the higher mean background OD450 and greater dispersion observed among the negative sera for this antigen compared with the B169L and KP177R assays, as the cut-off was calculated as the mean OD450 of the negative panel plus three standard deviations (Mean + 3SD). Despite the higher absolute threshold, the separation between positive and negative populations remained unambiguous: all 12 ASFV-positive sera yielded OD450 values clearly above the cut-off, whereas all 30 negative and all heterologous-pathogen sera fell below it, with no values in the borderline range. Nevertheless, we acknowledge that a cut-off derived from only 30 negative sera is preliminary. In future large-scale validation, cut-off values will be re-established on an expanded serum panel, and background reduction strategies, such as alternative blocking agents or pre-adsorption of sera with *E. coli* lysate, will be systematically evaluated for the A151R assay.

Despite the promising diagnostic performance demonstrated herein, this study has several important limitations. The validation serum panel was relatively small, geographically confined, and restricted to ASFV genotype II, thereby precluding large-scale field validation and cross-genotype or cross-serogroup assessment. This constraint is particularly pertinent given the recent emergence of genotype I and genotype I/II recombinant strains with distinct virulence profiles and genomic architectures in China and Asia. The ASFV-positive sera were certified reference materials obtained from experimentally infected domestic pigs, which, while ensuring standardization, limits extrapolation to naturally infected field animals and to other suid species such as wild boars. Future studies should therefore prioritize multi-center validation using well-characterized sera from diverse genotypes (genotypes I, II, and recombinants) and serogroups, alongside systematic benchmarking against commercially available diagnostic kits to establish relative field performance. Furthermore, although the present study focused on individual single-antigen ELISAs as a necessary foundational step, the development of multiplex detection platforms integrating multiple antigens represents a critical future direction. Such combinatorial formats were not pursued here because the primary objective was first to establish the baseline reactivity, optimal assay conditions, and individual diagnostic parameters for each novel antigen. Evaluation of assay sensitivity during early-stage infection prior to complete seroconversion, and the application of these assays for monitoring vaccine-induced humoral responses as candidate ASF vaccines advance toward regulatory evaluation, are also essential next steps. Finally, given that registered diagnostic kits often exhibit reduced sensitivity or specificity with wild boar sera, dedicated evaluation of these antigens using wild boar serum panels is warranted to assess their utility in sylvatic cycle surveillance.

## 5. Conclusions

This study identified and validated three novel ASFV recombinant antigens (B169L, A151R, and KP177R), demonstrating promising preliminary performance for serological detection within the tested serum. The developed indirect ELISAs exhibited preliminary specificity and reproducibility, with no cross-reactivity detected against the limited panel of antisera to major co-circulating porcine pathogens tested herein. Prokaryotic expression enables cost-effective and scalable antigen production, which may support broad implementation in resource-diverse settings following further validation. Together, these assays represent candidate tools with potential utility for ASF serological surveillance, epidemiological investigation, and clinical sample monitoring, pending large-scale, multi-center validation. Future research should focus on large-scale field validation across geographically and genotypically diverse sample populations, rigorous comparison with commercial diagnostic platforms, and the development of integrated multi-antigen detection systems to further enhance diagnostic sensitivity, coverage, and utility.

## Figures and Tables

**Table 1:** Amino acid sequence and nucleic acid sequence of the expressed constructs.

Protein	Expressed Region (aa)	Construct Description	Nucleotide Sequence (Codon-Optimized for *E. coli*)	Amino Acid Sequence	Molecular Mass + 6×His (kDa)
B169L	81–164	Encompassing the C-terminal proline-rich hydrophilic domain with the immunodominant epitopes.	catcaacttaacgacatctacaacaaaagcaacatggacgttattgttagcagcattcacgacaaatacaaaggcggtgacgaaattattcctcctatttcaccaccgtcagtttcaaacgaactggaggaagaccagccaaaaaaaaatcccggcaggtccgaaaccggcagacagcaaaccggtgagcctgcctgatagcaaaccgttagtcccgctgcaagaagttattatgccaagccaataataataac	HQLNDIYNKSNMDVIVSSIHDKYKGGDEIIPPISPPSVSNELEEDQPKKIPAGPKPADSKPVSLPDSKPLVPLQEVIMPSQYNN	16.87
A151R	50–110	Spanning the central thioredoxin-like core region.	aatcatgatctgataagcaaagaagatcttaaaggagcaacatccaaaaacattgctaaaatgatttataattggattataaaaaatcctcaaaataataagatttggagtggtgagccgcgtactcaaatttattttgaaaatgatttatatcatacaaattacaatcataaatgtata	DNHDLISKEDLKGATSKNIAKMIYNWIIKNPQNNKIWSGEPRTQIYFENDLYHTNYNHKCI	10.68
KP177R	38–177	Corresponding to the mature virion-associated p22 protein with the N-terminal signal peptide removed.	tgtaaagtagataaagattgtggtagtggagagcattgtgttcgtggatcatgtagctcattgagctgcttagatgccgtaaaaatggacaaacgaaatattaagatagattctaagatttcctcatgcgaattcactcccaatttttaccgttttacggatactgctgctgatgagcagcaagaatttggaaaaacacggcatcctataaaaataactccatctccaagtgaatcccatagcccccaagaggtgtgtgaaaaatattgttcatggggaaccgatgactgtacaggttgggaatatgttggtgatgaaaaggagggaacatgttatgtatataataatccacatcacccggttcttaaatatggtaaggatcacatcatagccttacctagaaatcataaacatgca	VCKVDKDCGSGEHCVRGSCSSLSCLLDAVKMDRNIKIDSKISSCEFTPNFYRFTTDTADEQQEFGKTRHPIKITPSPSESHSPQEVCEKYCSWGTDDCTGWEYVGDEKEGTCYVYNNPHHPVLKYGKDHIIALPRNHKHA	23.41

**Table 2:** Repeatability test of the three indirect ELISAs.

Assay	Serum Samples	ASFV-B169L		ASFV-A151R		ASFV-KP177R	
		Average Value	CV%	Average Value	CV%	Average Value	CV%
Intra- assay	No.P1	1.598	1.75	1.331	2.35	2.217	2.94
	No.P2	1.356	3.43	1.283	1.74	2.038	2.46
	No.P3	1.175	5.63	1.151	3.56	2.195	3.15
	No.N1	0.045	3.42	0.238	2.58	0.273	1.35
	No.N2	0.049	2.34	0.447	2.26	0.205	1.05
	No.N3	0.049	4.08	0.374	1.81	0.254	1.25
Inter- assay	No.P1	1.758	7.68	1.421	5.74	2.294	6.08
	No.P2	1.356	7.52	1.530	4.83	2.427	4.48
	No.P3	1.379	6.15	1.233	6.28	2.288	5.69
	No.N1	0.056	4.5	0.353	5.81	0.221	5.43
	No.N2	0.049	2.3	0.545	7.55	0.236	5.00
	No.N3	0.049	4.0	0.254	6.32	0.173	4.78

## Data Availability

The original contributions presented in this study are included in the article. Further inquiries can be directed to the corresponding authors.

## References

[1] Nguyen T., Kim Y.H., Nguyen V.D., Tran T., Vu N.D., Vu T., Nguyen V.T., Kim D.Y., Cho K.H., Hong S.K. (2026). Rapid Spread of Recombinant African Swine Fever Virus Genotypes I and II, Vietnam, 2023–2024. Emerg. Infect. Dis..

[2] Venturina V.M., Gundran R.S., Rafael R.B., Salvador R.T., Salinas M., Balagan E., Valdez P.M., Soriano A.P., Medina N.P., Garcia G.G. (2025). Surveillance and Management Strategies for African Swine Fever (ASF) in Central Luzon, Philippines. Pathogens.

[3] Franzoni G., Lean F., Giaconi E., Tedde G., Zinellu S., Nicolussi P., Le Dimna M., Le Potier M.F., Steedman E., Crooke H.R. (2026). Dynamics of leukocyte populations, immune-regulatory cytokines, and biochemical parameters in wild boar and domestic pigs experimentally infected with a virulent African swine fever virus genotype II strain. Front. Immunol..

[4] Skorobagatko D.A., Tolkova E.S., Shepeleva O.A., Shapovalov S.O. (2026). African swine fever virus (Asfarviridae, Asfivirus) strains from the central regions of Russia, carrying variant 5 of the central variable region (CVR), are characterized by tandem duplication in the intergenic region MGF 360-13L–MGF 360-14L. Vopr. Virusol..

[5] Friedrichs V., Schäfer A., Deutschmann P., Bock S., Hlinak A., Bock W.I., Moss A., Beer M., Blome S. (2026). Sensitivity and Specificity Assessment of Various African Swine Fever ELISA Kits for Accurate Detection of Seropositive Wild Boar. Pathogens.

[6] Ding H., Du L., Wang S., Zhong J., Zhang J., Li J., Zhang L., Zou X., Yang H., Zhang N. (2026). Ferritin nanoparticle-based indirect ELISA for immunodominant region screening and B-cell epitope validation of African swine fever virus. Int. J. Biol. Macromol..

[7] Luo M., Shuai W., Guo Z., Li J., Li L., Zhou Y., Jiang Y., Zeng Y., Wang J., Gao F. (2026). Indirect ELISA for African swine fever virus serological detection and recombinant porcine reproductive and respiratory syndrome virus-based bivalent vaccine. Int. J. Biol. Macromol..

[8] Wu P., McDaniel A.J., Rodríguez Y.Y., O’Donnell V., Jia W. (2026). A Novel In-Cell ELISA With Superior Sensitivity and Specificity for the Detection of African Swine Fever Virus-Specific IgM and IgG Antibodies. Transbound. Emerg. Dis..

[9] Lu H., Shao J., Liu W., Gao S., Guo H., Dong H., Zhou T., Yang J., Chang H. (2026). Identification of efficient multi-epitope combinations against African swine fever virus based on AP205 scaffold-mediated nanodisplay technology. J. Nanobiotechnol..

[10] Srionrod N., Wutthiwitthayaphong S., Nipakornpun T., Ruenphet S. (2026). High-Accuracy Serodiagnosis of African Swine Fever Using P72 and P30-Based Lateral Flow Assays: A Validation Study with Field Samples in Thailand. Vet. Sci..

[11] Tarigan S., Sumarningsih S., Ratnawati A., Saepulloh M., Wasito W., Sendow I., Nuradji H., Dharmayanti N. (2025). Development of a cost-effective serodiagnosis for African swine fever using solubility-enhanced recombinant p54, p30, and p72. Vet. World.

[12] Indrabalan U.B., Suresh K.P., Shivamallu C., Patil S.S. (2021). An extensive evaluation of codon usage pattern and bias of structural proteins p30, p54 and, p72 of the African swine fever virus (ASFV). VirusDisease.

[13] Jin J., Bai Y., Zhang Y., Lu W., Zhang S., Zhao X., Sun Y., Wu Y., Zhang A., Zhang G. (2023). Establishment and characterization of a novel indirect ELISA method based on ASFV antigenic epitope-associated recombinant protein. Int. J. Biol. Macromol..

[14] Gallardo C., Soler A., Nieto R., Carrascosa A.L., De Mia G.M., Bishop R.P., Martins C., Fasina F.O., Couacy-Hymman E., Heath L. (2013). Comparative evaluation of novel African swine fever virus (ASF) antibody detection techniques derived from specific ASF viral genotypes with the OIE internationally prescribed serological tests. Vet. Microbiol..

[15] Spinard E., Dinhobl M., Tesler N., Birtley H., Signore A.V., Ambagala A., Masembe C., Borca M.V., Gladue D.P. (2023). A Re-Evaluation of African Swine Fever Genotypes Based on p72 Sequences Reveals the Existence of Only Six Distinct p72 Groups. Viruses.

[16] Heimerman M.E., Murgia M.V., Wu P., Lowe A.D., Jia W., Rowland R.R. (2018). Linear epitopes in African swine fever virus p72 recognized by monoclonal antibodies prepared against baculovirus-expressed antigen. J. Vet. Diagn. Investig..

[17] Giménez-Lirola L.G., Mur L., Rivera B., Mogler M., Sun Y., Lizano S., Goodell C., Harris D.L., Rowland R.R., Gallardo C. (2016). Detection of African Swine Fever Virus Antibodies in Serum and Oral Fluid Specimens Using a Recombinant Protein 30 (p30) Dual Matrix Indirect ELISA. PLoS ONE.

[18] Ge B., Domesle K.J., Simmons J.A., Young S.R., Brake D.A., McDonald R.C., Yancy H.F., Gabbert L.R., Neilan J.G., Whitehouse C.A. (2025). Development of a novel p72 gene-based loop-mediated isothermal amplification assay for the rapid detection of African swine fever virus in animal feed. Front. Vet. Sci..

[19] Li J., Li Q., Wang Y., Guo Z., Qu Y., Wang X., Deng H., Dai J., Li L.F., He W.R. (2025). The B169L protein of African swine fever virus functions as a viroporin that activates the calcium-mediated inflammasome. PLoS Pathog..

[20] Ramirez-Medina E., Vuono E., Pruitt S., Rai A., Espinoza N., Valladares A., Spinard E., Silva E., Velazquez-Salinas L., Gladue D.P. (2022). ASFV Gene A151R Is Involved in the Process of Virulence in Domestic Swine. Viruses.

[21] Vuono E.A., Ramirez-Medina E., Pruitt S., Rai A., Espinoza N., Velazquez-Salinas L., Gladue D.P., Borca M.V. (2021). Evaluation of the Function of the ASFV KP177R Gene, Encoding for Structural Protein p22, in the Process of Virus Replication and in Swine Virulence. Viruses.

[22] Ren H., Wang Y., Li L.F., Shi L.F., Ma Y.H., Fan J.H., Pan X.Y., Shao H.C., Zhang Y., Han S. (2025). The African swine fever virus p22 inhibits the JAK-STAT signaling pathway by promoting the TAX1BP1-mediated degradation of the type I interferon receptor. PLoS Pathog..

[23] Neilan J.G., Zsak L., Lu Z., Burrage T.G., Kutish G.F., Rock D.L. (2004). Neutralizing antibodies to African swine fever virus proteins p30, p54, and p72 are not sufficient for antibody-mediated protection. Virology.

[24] Gómez-Puertas P., Rodríguez F., Oviedo J.M., Ramiro-Ibáñez F., Ruiz-Gonzalvo F., Alonso C., Escribano J.M. (1996). Neutralizing antibodies to different proteins of African swine fever virus inhibit both virus attachment and internalization. J. Virol..

[25] Li R., Peng Q., Li H., Zeng Z., Han T. (2025). Compound Interaction Presentation Learning for MHC-Peptide Binding Affinity Prediction. IEEE Trans. Comput. Biol. Bioinform..

[26] Liu Y., You G., Shi J., Gao L., Li X., Cao H., Wang Y., Zheng S.J. (2025). Indirect ELISA developed to detect antibodies against Mycoplasma synoviae P50 protein via immunoproteomic screening. Appl. Microbiol. Biotechnol..

[27] Raeisi H., Azimirad M., Schiopu A., Zarnani A.H., Asadzadeh Aghdaei H., Abdemohamadi E., Zali M.R., Yadegar A. (2025). Development of novel neutralizing single-chain fragment variable antibodies against S100A8. Sci. Rep..

[28] Waury K., Kvartsberg H., Zetterberg H., Blennow K., Teunissen C.E., Abeln S. (2025). Data-driven evaluation of suitable immunogens for improved antibody selection. Protein Sci..

[29] Li J., Jiao J., Liu N., Ren S., Zeng H., Peng J., Zhang Y., Guo L., Liu F., Lv T. (2023). Novel p22 and p30 dual-proteins combination based indirect ELISA for detecting antibodies against African swine fever virus. Front. Vet. Sci..

[30] Li Y., Huang L., Li H., Zhu Y., Yu Z., Zheng X., Weng C., Feng W.H. (2024). ASFV pA151R negatively regulates type I IFN production via degrading E3 ligase TRAF6. Front. Immunol..

[31] Guo S., Ru Y., Zhang H., Xue J., Liu H., Men D., Cui Z., Shen C., Tian H., Ma C. (2025). An African swine fever virus-specific antibody reactome reveals antigens as potential candidates for vaccine development. J. Virol..

[32] Alejo A., Matamoros T., Guerra M., Andrés G. (2018). A Proteomic Atlas of the African Swine Fever Virus Particle. J. Virol..

[33] Liu L., Chen Q., Yu L., Wu W., Mou S., Guo H., Tan C., Liu Q., Wang D., Chen H. (2026). Development of an indirect ELISA based on a multi-epitope recombinant protein for antibody detection against African swine fever virus. Microbiol. Spectr..

[34] Xu C., Liu H., Gu H., Tang X., Liang L., Hou S., Ding J., Zhao X., Liang R. (2026). Indirect ELISA Based on ASFV Polymerase Three Subunits for Serological Monitoring of African Swine Fever Antibodies. Vet. Sci..

